# Bias against research on gender bias

**DOI:** 10.1007/s11192-018-2667-0

**Published:** 2018-02-17

**Authors:** Aleksandra Cislak, Magdalena Formanowicz, Tamar Saguy

**Affiliations:** 1 Psychology Department, Nicolaus Copernicus University, Toruń, Poland; 2 Department of Psychology, University of Bern, Fabrikstrasse 8, 3012 Bern, Switzerland; 3 Psychology Department, Interdisciplinary Center (IDC) Herzliya, Herzliya, Israel

**Keywords:** Impact factor, Gender-science stereotype, Gender discrimination, Bibliometric analysis

## Abstract

The bias against women in academia is a documented phenomenon that has had detrimental consequences, not only for women, but also for the quality of science. First, gender bias in academia affects female scientists, resulting in their underrepresentation in academic institutions, particularly in higher ranks. The second type of gender bias in science relates to some findings applying only to male participants, which produces biased knowledge. Here, we identify a third potentially powerful source of gender bias in academia: the bias against research on gender bias. In a bibliometric investigation covering a broad range of social sciences, we analyzed published articles on gender bias and race bias and established that articles on gender bias are funded less often and published in journals with a lower Impact Factor than articles on comparable instances of social discrimination. This result suggests the possibility of an underappreciation of the phenomenon of gender bias and related research within the academic community. Addressing this meta-bias is crucial for the further examination of gender inequality, which severely affects many women across the world.

## Introduction

Science it would seem is not sexless; she is a man, a father and infected too. (Woolf 1938/2015, p. 212)

Since Virginia Woolf made this statement, many changes have transpired. Gender bias in academia and science has been acknowledged (European Commission 2016a; Francisco 2007; Ritz et al. 2014), and intense measures have been taken to overcome it (for example, the National Science Foundation’s ADVANCE program aiming to increase the number of female scientists in academic STEM careers). However, despite the gender equality policies implemented in academia and society, the chances of women achieving professorships have hardly improved over the past 20 years (Danell and Hjerm 2013). Not surprisingly, women continue to be underrepresented in academic institutions, especially in higher ranks and decision-making bodies (European Commission 2016b) and in editorial boards (Cho et al. 2014). Women are also paid less than men (Shen 2013), and their research is less likely to receive funding (Ley and Hamilton 2008). Importantly, these differences cannot be attributed to levels of commitment or achievement (Wennerås and Wold 1997). In a study comparing promotion patterns, it was established that despite the similar productivity of men and women at the level of associated professor, men were still more likely to get promoted to full professorships (Carter et al. 2017). As such, it is likely that the gender gap in academia is due to the perceived lack of fit between scientific roles and being a woman (Eagly 1987; Smyth and Nosek 2015). Accordingly, it is ten times more common for female authors to be incorrectly cited as males than for male researchers to be misattributed as female (Krawczyk 2017). Similarly, studies have shown that when all other factors were held constant, women were less likely to be hired than men for the role of lab manager (Moss-Racusin et al. 2012) and that their research was devalued (Larivière et al. 2013).

Another type of gender bias in science relates to scientific investigations. As scientific inquiries often disregard the moderating roles of sex or gender, some findings apply mostly to male participants, producing biased knowledge. This can be detrimental, as findings pertinent to men may be irrelevant and, in the worst cases, harmful for women. For example, women’s underrepresentation as subjects in medical research may have grave consequences for women’s health (Correa-de-Araujo 2006).

In this research we focus on yet another type of gender bias in science, one that pertains to the very topics of scientific inquires. Particularly, we investigated a bias that is directed toward scientific efforts devoted to exposing and probing the phenomena of gender bias. We aim to verify whether gender bias as a topic of scientific investigation may be a subject of a biased evaluation resulting in fewer and less prestigious publications and fewer funding opportunities. Recent evidence suggests that men, relative to women, judge studies on gender discrimination less favorably (Handley et al. 2015). We here extend this evidence in important ways. First, by employing a bibliometric analysis we focus on outcomes that go beyond self-report ratings of valence to reflect consequential outcomes as they naturally occur in the field. Second, we consider the real-life scientific evaluations of research on gender bias in comparison to the analogous evaluations of research on an equivalent instance of social bias (for the two other biases toward female scientists and participants, the point of comparison was their male counterparts). Here, research on race bias constitutes a suitable point of reference. First, both race and gender are the most salient social categories and are both categorized in the early stages of information processing (Ito and Urland 2003). Related to this, discrimination based on these two categories forms the most recognizable and identifiable cases of social injustice, and, most likely for that reason, they are both named first in international documents referring to human rights. Finally, these two biases are investigated within the same disciplines, with researchers being trained in the same principles and methods and, by consequence, targeting similar choices for grant and publication outlets. Moreover, as men and women undertake similar research topics within psychology (König et al. 2015), it is likely that the gender composition of the team dealing with each topic is balanced.

Considering that no discrimination is less or more important than any other form of discrimination and that both biases are investigated with the same methodology, the bias against the examination of gender bias shall have no objective reason. One potential argument against studying gender discrimination could be bias-driven itself, stereotyping gender topics as “feminine,” and, as such, less valuable (Williams et al. 2010) and perceived as relatively incompetent and unscientific (Crawley 2014; Eagly 1987; Nosek et al. 2009). As competence is especially important for the evaluation of scientific merit (Madera et al. 2009), women-oriented research can seem less persuasive.

Another possible reason for disregarding gender over race in social science could be related to the availability heuristic (Tversky and Kahneman 1974). When looking at the conference halls or the departmental staff of many universities (not taking rank into account), at least half of the crowd are women (National Science Foundation 2015). Moreover, everybody can provide an example of a woman who has succeeded in her career or an example of real or perceived gender equality from their own house, neighborhood, or office. With this anchor, it appears that gender equality has already been achieved, and this might be sufficient to refute the phenomenon of gender bias or, at least, limit its potential impact. Race bias, however, provides many fewer viable examples. In science and in politics, there are still large discrepancies between the numbers of black and white scholars (National Center for Education Statistics 2014; Hopkins et al. 2013), and inter-racial contacts are far less frequent than inter-gender ones (Ridgeway and Smith-Lovin 1999). Following the logic of the availability heuristic, this might lead to conclusions of stronger (or more real) discrimination.

Drawing on these ideas, we propose that studying gender bias may meet with lower appreciation within the scientific community in comparison to studying race bias. We predict that this manifests in relatively lower appreciation in peer-review outcomes, reflected in less grant funding and fewer publications in prestigious journals for research on gender bias. Our goal in the current research was to provide the first evidence for this hypothesis by means of a bibliometric analysis. We compared articles that refer to gender bias to articles published on racial bias in terms of two types of peer-review outcomes: their Impact Factor and having grant support.

## Method

### Sample of articles

Using the EBSCO search engine, we retrieved all the peer-reviewed articles that used the keywords *gender bias*; *gender discrimination*, *gender prejudice and sexism versus race/racial bias*; *race/racial discrimination*, and *race/racial prejudice and racism* in their titles and that were listed in PsycINFO and PsycARTICLES databases covering 2534 journals. The timeframe of publication was set to 2008–2015. This timeframe choice was dictated by the availability of Impact Factor indexes through the library services accessible to the authors. Yet, this timeframe choice allowed the identification of more than half of all the articles that were ever published and matched the search criteria (prior to 2008, *N* = 1445; in years 2008–2015, *N* = 1485). As the shares of gender- and race-related issues were virtually the same in both periods of time (31.65% of articles published prior to 2008 and 31.71% of articles published between 2008 and 2015 referred to gender bias), we consider the chosen sample a representative sample of articles on race and gender bias/prejudice/discrimination. We identified 1485 articles published in 520 journals. Each article was assigned a numerical value based on the type of bias it referred to (race = 0; gender = 1). The full dataset with all the identified articles can be found in the Supplemental Online Materials (https://osf.io/y9tfv/).

Duplicates that were not automatically removed were manually removed (43 duplicates and 5 reprints). Additionally, we removed articles loading in two categories (52 articles had both gender and race terms in their titles), 1 non-English article, 9 errata, and 1 retracted article and its retraction note. Abstracts were inspected, and 39 articles regarding instances of non-social discrimination or bias (such as articles regarding own-gender or own-race bias in facial discrimination) were excluded.

Moreover, given the focus of the investigation on research on gender bias, we included in the database empirical articles using both qualitative and quantitative methodologies. Articles were identified as empirical or not through the PsycINFO meta-information. On rare occasions in which the PsycINFO meta-information was missing, the two first authors listed for this paper reached a decision by consensus. In total 282 articles were identified as non-empirical, such as commentaries, interviews, letters, literature reviews (but not meta-analyses), editorials, and book reviews. Given that such articles do not report on original research, often follow a different review process, and are seldom the basis of a grant application, we did not include them in the final sample. The information for four articles was missing, so they were also not included in the final sample.

### Impact factor and funding

We chose two peer-review criteria. The first was Impact Factor. Despite debates on whether Impact Factor values reflect the quality of science published in journals (Garfield 1996), it is still commonly recognized as reflecting the prestige of the journals, scientific impact, and broader public outreach (Perneger 2010). Each article was assigned a five-year Impact Factor value obtained for the respective journal from the Web of Science. In 185 cases, the Impact Factor indicators were unavailable for the year the selected articles were published. The five-year Impact Factor values ranged from .23 to 31.05 (*M* = 2.47; *SD* = 2.14). The preliminary analyses showed that the distribution of the five-year Impact Factor values was strongly skewed, and we identified two outliers with z-scores equal to 13.38. These outliers were articles published in *Science*. As this journal clearly belonged to a different population of top journals (which are unusually highly cited and in which acceptance decisions are made using different criteria than in other general journals), these observations were excluded from the analysis. Both of the excluded papers referred to race bias. The second indicator of peer review was information on whether the selected article was supported by funding (0 = no; 1 = yes). This information was available from the PsycINFO and PsycARTICLES databases.

### Journals and methods

We acknowledge that articles on gender/race bias might be sent not only to general interest journals but also to journals devoted to examining either gender or race inequalities. In general interest journals, articles on gender/race bias compete with all the other articles. Notably, research probing gender phenomena is published by journals with lower impact factors when it is explicitly labeled as “gender studies,” thus seemingly narrowing the scope of the article (Madison et al. 2016; see also Lundgren et al. 2015). In specialized journals, the publication of an article with merit that is aligned with a journal’s scope might be relatively easier. To control for that possibility, we included an additional coding that indicated whether the article was published in a general interest or specialized journal. One hundred fifteen papers on gender bias were published in 15 specialized journals dedicated to gender issues, such as *Gender & Society*, *Sex Roles*, or *Psychology of Women Quarterly*. One hundred fourteen papers on race bias were published in 25 specialized journals, of which 13 were dedicated to race issues, such as *Ethnic and Racial Studies*, *Journal of Black Psychology*, *Journal of Black Studies*, or *Race and Social Problems*, and 12 to gender issues, such as *European Journal of Women’s Studies* or *Women’s Studies International Forum*. The general summary of articles published on gender and race is presented in Table 1.

**Table 1:** General descriptive statistics for articles on gender and race bias

Type of bias	Number of articles	Number of funded articles	Number of articles in general journals	Mean 5-year IF (SD)	Mean % of female authors (SD)
Gender	355	142	240	2.09 (1.31)	66.42 (33.01)
Race	691	314	577	2.58 (1.77)	56.31 (36.20)

Furthermore, for all the empirical papers, the methodologies employed in the studies either qualitative or quantitative were recorded. The latter classification was based on the possibility that research on gender bias might be done using qualitative methods due to a feminist tradition that questions the objectivity of quantitative methods (Eagly and Riger 2014). As most of the journals cited within the scope of this bibliometric analysis predominantly publish papers using quantitative methodology (Eagly and Riger 2014), and given that qualitative research may be less funded than quantitative research (Carey and Swanson 2003; Morse 1999), controlling for the method used in the studies is crucial in establishing whether or not bias against research on gender bias is an artifact of the type of methodology possibly preferred in gender bias research. The summary of the empirical articles published on gender and race for papers using either qualitative or quantitative methods is presented in Table 2.

**Table 2:** Descriptive statistics for empirical articles on gender and race bias presented for papers employing either qualitative or quantitative methods

	Qualitative articles					Quantitative articles				
	Number of articles	Number of funded articles	Number of articles in general journals	Mean 5-year IF (SD)	Mean % of female authors (SD)	Number of articles	Number of funded articles	Number of articles in general journals	Mean 5-year IF (SD)	Mean % of female authors (SD)
Gender	27	7	22	1.63 (.86)	84.04 (29.09)	328	135	218	2.12 (1.33)	65.02 (32.94)
Race	176	51	139	1.45 (.81)	62.37 (41.92)	515	263	438	2.92 (1.83)	54.20 (33.78)

### Authorships

In order to account for bias against female authors, we also considered the gender composition of the research teams. To that end, we first determined each author’s gender by the first name’s gender stereotypicality or through a web search of departmental or private webpages, pictures, or other on-line records. In the case of twelve articles we could not assign gender of the authors due to lack of sufficient records.

As the rules regarding the relationship between the order of authors’ names and their relative input vary across disciplines (in some disciplines, the norm is to arrange authors’ names in an alphabetical order; in other disciplines, the most prestigious authorship position can be first or last), we decided to use the authorship gender indicator that would not be directly affected by a discipline’s rules in our analysis. Following Naldi and Parenti (2002; see also Kretschmer et al. 2012), we computed the *Contribution* bibliometric indicator—the percentage of women in each author team.

## Results

In order to examine the existence of bias against gender bias as a scientific topic, we conducted a regression analysis with two indicators of peer review as dependent variables. As predictors, we used the type of bias studied and the percentage of women in the research teams. All the correlation coefficients are listed in Table 3.

**Table 3:** Correlation coefficients for the variables used in the study

Variable	2	3	4	5	6
1. Type of bias (0 = race; 1 = gender)	− .05^	− .14***	.13***	.21***	− .18***
2. Grant (0 = no; 1 = yes)		.25***	− .03	.15***	− .001
3. 5 − year IF			− .09*	.26***	.26***
4. % of Female authors				− .07*	− .13***
5. Type of method (0 = Qual; 1 = Quant)					− .01
6. Type of journal (0 = specialty; 1 = general)					

^*p* = .10; *p < .05; **p < .01; ***p < .001

It is important to note that the cases were non-independent because some papers were published by the same journals, and this implies similar editorial policies and contingent five-year Impact Factor values. Therefore, in the final analyses, conducted using the MPlus program (Muthén and Muthén 2012), we nested papers in journals to obtain a robust standard error estimation.

**Fig. 1:**
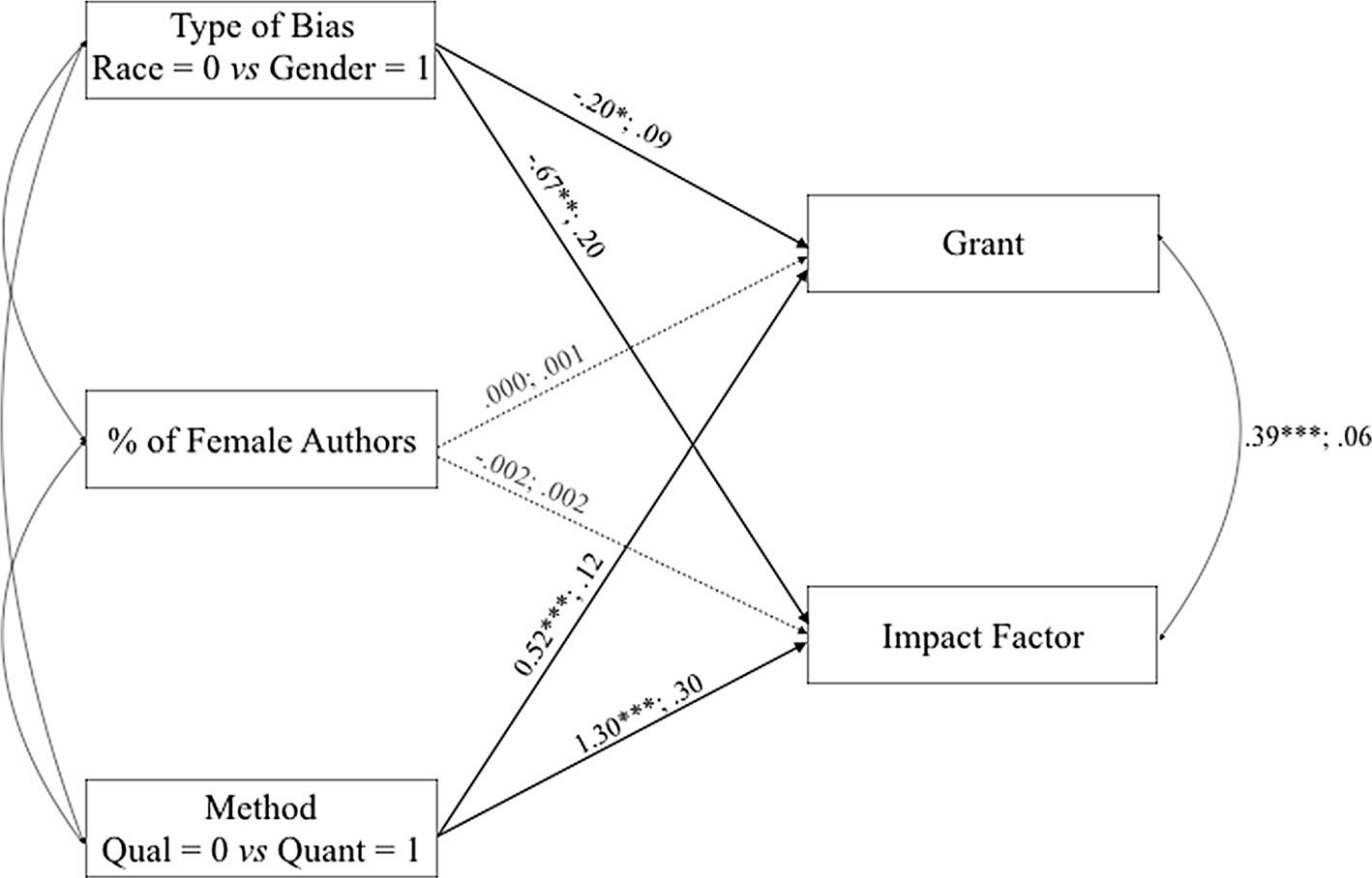
The tested model involving grant funding and Impact Factors as dependent variables and type of bias, percentage of female authors, and method as predictors. The first number represent an unstandardized coefficient, followed by its standard error. Dotted lines represent non-significant paths

As illustrated in Fig. 1, research on gender bias was funded less often (*B* = − .20; *SE* = .09; *p* = .02) and published in lower Impact Factor journals (*B* = − .67; *SE* = .20; *p* = .001). Importantly, including the percentage of females in the research teams as a covariate in the model showed that this percentage was not significantly related to financial support (*p* = .76) or to the prestige of the journal (*p* = .35). When those two nonsignificant parameters were constrained to 0, the final model fitted the data very well, *χ*^2^ (2) = 1.81; *p* = .40.

In an additional analysis, we have added the type of journal (general versus specialized) as a covariate. Yet, the inclusion of a covariate did not affect the predicted results pattern. The type of bias remained a significant predictor of both funding and Impact Factor. When we used the gender of the first author instead of the percentage of female authors as a predictor, interestingly, papers with women as first authors had a higher likelihood of being funded (*B* = .19; *SE* = .08; *p* = .02). Yet, the pattern of the role of methodology and, more importantly, the topic of investigation remained unchallenged.

## Discussion

Articles on gender discrimination were funded less often and published in less prestigious journals than papers on racial discrimination. Considering that the percentage of women in the author pool had a virtually null effect on both grant acquisition and the Impact Factor, this effect cannot be explained in terms of the gender of the researchers. So, what are the possible causes of this effect? Although we controlled for the type of methods used in the studies in the analysis, we cannot rule out the possibility that the articles on gender bias were of a different quality than the articles on race bias. This is, however, unlikely for the following reasons. First, as mentioned before, both gender and race bias are investigated within the same fields using similar methods and paradigms. It is rather unlikely that researchers who deal with gender research are less experienced or were trained in worse schools than researchers on race bias. Even less likely is that they used flawed methodology on purpose. Moreover, all the analyzed articles passed peer-review screening, which is considered a quality watchdog within the scientific community. Finally, the Impact Factor index is not a measure of the quality of individual research articles. As indicated in the San Francisco Declaration of Assessment of Research, it shall not be used as such, nor to assess individual scientists’ contributions or in hiring, promotion, or funding decisions. The Impact Factor was introduced to help librarians make decisions about popular journals within the field (Garfield 1996). All of the above suggest that bias against research on gender bias is not merit based but a reflection of a topic’s lower prestige and appreciation due to a generalized gender bias (as outlined in the introduction).

Another potential alternative explanation for the observed differences in grant funding for racial and gender bias research is the relative difference in the availability of participant samples. Recruiting racially diverse samples may be more difficult, time-consuming, and costly, while recruiting gender-diverse samples may be less of a problem, thereby necessitating less funding for recruitment. Future research should probe whether researchers studying gender bias are less eager to apply for funding or the gender bias applications are less likely to be funded, mirroring the effect of a female grant authorship (Ley and Hamilton 2008). It seems less plausible, though, that the availability of participant samples may affect the teams’ decisions regarding the outlet for their work. Admittedly, the current bibliometric analysis does not allow for the determination of whether the research programs on gender bias are submitted to (and, as a consequence, also published by) less prestigious journals or are rejected by more prestigious journals. If the first explanation is correct, it either implies that researchers are aware of the existence of bias against gender bias research among academic community or that they consider their own work less suitable for more prestigious journals, thus perpetuating the existing arrangements. The second mechanism—the rejection by more prestigious journals—would be in line with previous research on the existence of subtle bias in the perceived quality of studies evidencing gender discrimination (Handley et al. 2015). Given that abstracts on gender bias research were evaluated less positively by male reviewers (Handley, et al. 2015), one possible explanation for the obtained result is that the research on gender bias is more often reviewed by male researchers than research on race bias. With the available dataset, we cannot rule out that possibility. However, considering that the pool of reviewers, due to common editorial strategies, is likely to be similar to the author pool (for gender research, comprising 66% women, and for race research, 56% women) this seems rather unlikely. Nevertheless, future research could examine whether the type of discrimination (gender versus race) is sent to reviewers of different genders and whether this relationship varies by the journal’s prestige.

### Implications

The reported bias is a potentially powerful phenomenon because it might be invisible to the scientific community and, as such, overlooked (Blair et al. 2004; Carnes et al. 2012; Devine et al. 2017) and not taken care of (in contrast to the first and second types of bias mentioned in the introduction, for which corrective measures have been applied). By raising the awareness of the existence of bias against research on gender bias among male researchers (Handley et al. 2015) and in general as a topic of scientific inquiry, as in our study, we hope to contribute to the discussion about the fairness of journal policies. This discussion is primarily important in order for gender bias to be properly acknowledged within the scientific community and to pursue further examination of this powerful source of inequality that severely affects many women in the world (UN Women 2015). Gender bias intersects with social standing, resulting in a feminization of poverty (Starrels et al. 1994). In addition, it intersects with race bias in academic communities (Gutiérrez y Muhs et al. 2012; Hopkins et al. 2013) and elsewhere (Livingston et al. 2012; United Nations Division for the Advancement of Women [DAW], Office of the High Commissioner for Human Rights [OHCHR], United Nations Development Fund for Women [UNIFEM] 2000). Finally, addressing this meta-bias within academia will strengthen the scientific community and its ability to expose and overcome bias and to maintain its powerful ability to inform organizational, national-level, and international policies and decision-making processes.

## References

[CR1] Blair IV, Judd CM, Chapleau KM (2004). The influence of Afrocentric facial features in criminal sentencing. Psychological Science.

[CR2] Carey MA, Swanson J (2003). Funding for qualitative research. Qualitative Health Research.

[CR3] Carnes M, Devine PG, Isaac C, Manwell LB, Ford CE, Byars-Winston A, Sheridan J (2012). Promoting institutional change through bias literacy. Journal of Diversity in Higher Education.

[CR4] Carter TE, Smith TE, Osteen PJ (2017). Gender comparisons of social work faculty using h-index scores. Scientometrics.

[CR5] Cho AH, Johnson SA, Schuman CE, Adler JM, Gonzalez O, Graves SJ, Bruna EM (2014). Women are underrepresented on the editorial boards of journals in environmental biology and natural resource management. PeerJ.

[CR6] Correa-de-Araujo R (2006). Serious gaps: How the lack of sex/gender-based research impairs health. Journal of Women’s Health.

[CR7] Crawley D (2014). Gender and perceptions of occupational prestige: Changes over 20 years. SAGE Open.

[CR8] Danell R, Hjerm M (2013). Career prospects for female university researchers have not improved. Scientometrics.

[CR9] Devine PG, Forscher PS, Cox WTL, Kaatz A, Sheridan J, Carnes M (2017). A gender bias habit-breaking intervention led to increased hiring of female faculty in STEMM departments. Journal of Experimental Social Psychology.

[CR10] Eagly AH (1987). Sex differences in social behavior: A social-role interpretation.

[CR11] Eagly AH, Riger S (2014). Feminism and psychology: Critiques of methods and epistemology. American Psychologist.

[CR12] European Commission. (2016a). *Horizon 2020. Promoting gender equality in research and innovation.* Retrieved from: https://ec.europa.eu/programmes/horizon2020/en/h2020-section/promoting-gender-equality-research-and-innovation

[CR13] European Commission. (2016b). *She figures 2015* [online report]. Retrieved from: https://ec.europa.eu/research/swafs/pdf/pub_gender_equality/she_figures_2015-final.pdf

[CR14] Francisco, J. G. (2007). *Summary report: Women’s studies/gender research meeting* [online report]. Retrieved from: http://www.unesco.org/fileadmin/MULTIMEDIA/HQ/SHS/pdf/Summary-Report-QuezonCity.pdf

[CR15] Garfield E (1996). Fortnightly review: How can impact factors be improved?. BMJ.

[CR16] Gutiérrez y Muhs GG, Niemann YF, González CG, Harris AP (2012). Presumed incompetent: The intersections of race and class for women in academia.

[CR17] Handley IM, Brown ER, Moss-Racusin CA, Smith JL (2015). Quality of evidence revealing subtle gender biases in science is in the eye of the beholder. Proceedings of the National Academy of Sciences.

[CR18] Hopkins AL, Jawitz JW, McCarty C, Goldman A, Basu NB (2013). Disparities in publication patterns by gender, race and ethnicity based on a survey of a random sample of authors. Scientometrics.

[CR19] Ito TA, Urland GR (2003). Race and gender on the brain: Electrocortical measures of attention to the race and gender of multiply categorizable individuals. Journal of Personality and Social Psychology.

[CR20] König CJ, Fell CB, Kellnhofer L, Schui G (2015). Are there gender differences among researchers from industrial/organizational psychology?. Scientometrics.

[CR21] Krawczyk M (2017). Are all researchers male? Gender misattributions in citations. Scientometrics.

[CR22] Kretschmer H, Kundra R, Beaver D, Kretschmer T (2012). Gender bias in journals of gender studies. Scientometrics.

[CR23] Larivière V, Ni C, Gingras Y, Cronin B, Sugimoto CR (2013). Bibliometrics: Global gender disparities in science. Nature.

[CR24] Ley TJ, Hamilton BH (2008). The gender gap in NIH grant applications. Science.

[CR25] Livingston RW, Rosette AS, Washington EF (2012). Can an agentic Black woman get ahead? The impact of race and interpersonal dominance on perceptions of female leaders. Psychological Science.

[CR26] Lundgren S, Shildrick M, Lawrence D (2015). Rethinking bibliometric data concerning gender studies: A response to Söderlund and Madison. Scientometrics.

[CR27] Madera JM, Hebl MR, Martin RC (2009). Gender and letters of recommendation for academia: Agentic and communal differences. Journal of Applied Psychology.

[CR28] Madison G, Söderlund T (2016). Can gender studies be studied? Reply to comments on Söderlund and Madison. Scientometrics.

[CR29] Morse JM (1999). Qualitative generalizability. Qualitative Health Research.

[CR30] Moss-Racusin CA, Dovidio JF, Brescoll VL, Graham MJ, Handelsman J (2012). Science faculty’s subtle gender biases favor male students. Proceedings of the National Academy of Sciences.

[CR31] Muthén LK, Muthén BO (2012). *MPlus. Statistical analysis with latent variables user’s guide*.

[CR32] Naldi, F., & Parenti, I.V. (2002). *Scientific and technological performance by gender: A feasibility study on patent and bibliometric indicators. Volume II: methodological report*. European Commission Research, EUR 20309.

[CR33] National Center for Education Statistics. (2014). *Postsecondary education in digest of education statistics 2014, chapter 3*. National Center for Education Statistics, U.S. Department of Education

[CR34] National Science Foundation. (2015). *NSF/NIH/USED/USDA/NEH/NASA 2013 Survey of Earned Doctorates, special tabulation*. National Center for Science and Engineering Statistics.

[CR35] Nosek BA, Smyth FL, Sriram N, Lindner NM, Devos T, Ayala A, Greenwald AG (2009). National differences in gender-science stereotypes predict national sex differences in science and math achievement. Proceedings of the National Academy of Sciences.

[CR36] Perneger TV (2010). Citation analysis of identical consensus statements revealed journal-related bias. Journal of Clinical Epidemiology.

[CR37] Ridgeway CL, Smith-Lovin L (1999). The gender system and interaction. Annual Review of Sociology.

[CR38] Ritz SA, Antle DM, Cote J, Deroy K, Fraleigh N, Messing K, Mergler D (2014). First steps for integrating sex and gender considerations into basic experimental biomedical research. The FASEB Journal.

[CR39] Shen H (2013). Inequality quantified: Mind the gender gap. Nature.

[CR40] Smyth FL, Nosek BA (2015). On the gender–science stereotypes held by scientists: Explicit accord with gender-ratios, implicit accord with scientific identity. Frontiers in Psychology.

[CR41] Söderlund T, Madison G (2015). Characteristics of gender studies publications: A bibliometric analysis based on a Swedish population database. Scientometrics.

[CR42] Starrels ME, Bould S, Nicholas LJ (1994). The feminization of poverty in the United States: Gender, race, ethnicity, and family factors. Journal of Family Issues.

[CR43] Tversky A, Kahneman D (1974). Judgment under uncertainty: Heuristics and biases. Science.

[CR44] UN Women. (2015). *Progress of the world’s women 2015*–*2016: Transforming economies, realizing rights*. United Nations Division for the Advancement of Women (DAW), Office of the High Commissioner for Human Rights (OHCHR), United Nations Development Fund for Women (UNIFEM). (2000). *Gender and racial discrimination report of the expert group meeting*.

[CR45] Wennerås C, Wold A (1997). Nepotism and sexism in peer-review. Nature.

[CR46] Williams MJ, Levy Paluck E, Spencer-Rodgers J (2010). The masculinity of money: Automatic stereotypes predict gender differences in estimated salaries. Psychology of Women Quarterly.

[CR47] Woolf, V. (1938/2015). *A room of one’s own. Three guineas*. Oxford: Oxford University Press.

